# Interferon autoantibodies as signals of a sick thymus

**DOI:** 10.3389/fimmu.2024.1327784

**Published:** 2024-02-22

**Authors:** Bergithe E. Oftedal, Thea Sjøgren, Anette S. B. Wolff

**Affiliations:** ^1^Department of Clinical Science, University of Bergen, Bergen, Norway; ^2^Department of Medicine, Haukeland University Hospital, Bergen, Norway

**Keywords:** type I interferons, autoimmunity, immune deficiencies, thymus, AIRE

## Abstract

Type I interferons (IFN-I) are key immune messenger molecules that play an important role in viral defense. They act as a bridge between microbe sensing, immune function magnitude, and adaptive immunity to fight infections, and they must therefore be tightly regulated. It has become increasingly evident that thymic irregularities and mutations in immune genes affecting thymic tolerance can lead to the production of IFN-I autoantibodies (autoAbs). Whether these biomarkers affect the immune system or tissue integrity of the host is still controversial, but new data show that IFN-I autoAbs may increase susceptibility to severe disease caused by certain viruses, including SARS-CoV-2, herpes zoster, and varicella pneumonia. In this article, we will elaborate on disorders that have been identified with IFN-I autoAbs, discuss models of how tolerance to IFN-Is is lost, and explain the consequences for the host.

## Thymic development and function

The thymus is a primary lymphoid organ located in the thoracic cavity, behind the sternum and above the heart. It develops together with the parathyroid glands from the most anterior region of the foregut, the pharynx, from which they bud out and detach, before migrating to their final location. The thymus holds a highly specialized microenvironment with a unique capacity to support efficient development of self-tolerant T cells expressing a wide repertoire of T cell receptors (antigen receptors) (1–3).

The thymus has two equal lobes consisting of a cortex and a medulla. The cortical-medullary junction intersects these regions and contains the blood vessels responsible for both transporting hematopoietic progenitors from the bone marrow into the thymus, and mature T lymphocytes out of the thymus to peripheral lymphoid organs (1, 4). The cortex is the site of early-thymocyte development and is filled with double-positive thymocytes. Single-positive thymocytes are found in the medulla where late-thymocyte development occurs before they egress from the thymus (5–7). T cell differentiation and development has been extensively reviewed elsewhere (8–10). In addition to the mesenchymal, endothelial, and dendritic cells required for efficient T cell development, both compartments contain distinct populations of thymic epithelial cells (TECs) (1, 11, 12). TECs create a three-dimensional network in which thymocytes migrate through their development. The correct patterning and organization of the thymic stromal components are essential for optimal T cell development and thymus function. Thymic cross talk, the interactions between thymocytes and TECs, is required in the formation of the stromal microenvironment and likely influences both the cortex and the medulla (8, 9). Structural or developmental thymic defects can result in serious health consequences, including immunodeficiency and autoimmunity (1).

The importance of correct thymic development is illustrated by how loss of function (LOF) mutations in FOXN1 (forkhead family transcription factor), the main transcription factor required for correct TEC proliferation and differentiation, can cause immune related diseases. Nude mice deficient in Foxn1 have thymic abnormalities and they develop alopecia universalis and nail dystrophy (13–17). In humans, FOXN1 deficiency leads to a rare form of severe combined immunodeficiency (SCID) (18) with absent or low T cell numbers due to abnormal thymic stroma (18) (19). Abnormal thymic stromal phenotypes are associated with various inherited autoimmune diseases in humans or their disease-related mouse models, as seen in **Table 1**. Another important protein for thymic immune tolerance is the Autoimmune Regulator (AIRE), which regulates the expression of a panel of tissue-restricted antigens (TRAs) to be presented to developing T cells, in medullary TECs (mTECs) (95, 96). AIRE deficiency leads to autoimmune polyendocrine syndrome type I (APS-I), a rare disease characterized by endocrine autoimmunity and chronic mucocutaneous candidiasis (97). Other transcription factors with roles complementary to AIRE might exist to secure immune tolerance to a broader range of TRAs. Although FEZ Family Zinc Finger 2 (FEZF2) has been suggested as one candidate (98, 99), no human disease is directly linked to mutations in this gene. Other disorders that implicate disrupted thymic organization, and thereby incomplete central tolerance, include hypomorphic *RAG 1* and *2* mutations (Omenn syndrome) (37), resulting in failure of variable (V), diversity (D), and joining (J) segment recombination in the generation of the highly diverse B and T cell receptors; DiGeorge syndrome (76) where patients have microdeletions of chromosome 22; Down syndrome with trisomy 21 (76), and the polygenic disorder systemic lupus erythematosus (SLE) (65, 66) (**Table 1**).

**Table 1:** Diseases/groups seen with interferon type 1 autoantibodies: genetic determinants and their consequences when mutated.

Gene/ Protein	Type of mutation	Affected Thymus	Disease name/Phenotype#, OMIM	MI	Autoimmunity	Immune deficiency	Affected IFN-I (blood)	IFN-I Abs	Affected AIRE	Affected Tregs	References
AIRE	LOF	x	APS-I 240300	AD, AR	Endocrine, ectodermal, Enteropathy	C. albicans, Severe Covid-19, herpes zoster	Down	Yes (α,ω)^&^	Down	Yes	(20–24)
FOXP3	LOF	(x)	IPEX 304790	XLR	T1D, enteropathy, other	Skin		Yes		Yes	(25, 26)
NFKB2	LOF	X	DAVID 615577	AD	Diverse autoimmunity	CVID, hypogammaglubolinemia		Yes	Down*	No (mice)	(27–30)
RELB	LOF	(x)	617585	AR	Diverse autoimmunity	CVID		Yes	Down*	No (mice)	(31, 32)
IKBKG (NEMO/ IKK- γ)	LOF	(x)	BSS; epilepsy 300248	XLR, XLD	Hemolytic anemia; thrombocytopeni; colitis	CVID	Up	Yes		Yes	(33–36)
RAG1/2	Hypo morphic	X	Omenn 603554	AR	Diverse autoimmunity	SCID, Leaky SCID		Yes	Down	Yes	(37–41)
STAT1	GOF	X	600555	AD, AR	Diverse autoimmunity	CVID, (C.albicans)	Up	Yes		No	(27, 42–46)
CTLA4	LOF	(x)	123890	AD	Enteropathy, cytopenia, thyroiditis, other	Diverse immune deficiency		Yes		Yes	(27, 47)
IKZF2/ HELIOS	LOF	(x)	606234	AD, AR	Endocrine¤, other	CVID, IgA-deficiency¤		Yes		Yes	(27, 48)
LCK	LOF?	(x)	HP¤, skin, gut 615758	AR	Diverse autoimmunity	CVID		Yes¤		Yes	(27, 49, 50)
LAT	LOF?	(x)	602354	AR	Diverse autoimmunity, endocrine¤	CVID; IgA-deficiency¤		Yes¤		Yes	(27, 51–53)
TNFAIP3/ A20¤	LOF?	(x)	HA20 191163	AD	Autoinflammatory; Behcet’s; endocrine¤, ulcers	CVID; IgA-deficiency¤,	Up	Yes¤		Yes	(27, 54–57)
JAK3	LOF?	(x)	A dysfunc. 600173	AR	Enteropathy	SCID, skin		Yes		Yes	(27, 58)
HLA+?		x	MG and thymoma 254200		Muscles, thymus	With thymoma: C.albicans	Up	Yes (α,ω)^&^	Down	Yes	(59–64,)
Polygenic		x	SLE; Fluct. 152700		Autoinflammatory, SLE		Up	Sub-cohorts		Yes (number)	(60, 65–73)
Polygenic			RA; Fluct. 180300		Autoinflammatory, RA		Up	Sub-cohorts		Contradictory	(67, 73–75)
21q22.3		x	Down Syndrome 190685	IC	Endocrine		Down	Minor	Down	Yes	(76–79)
22q11.2		x	DiGeorge Syndrome 18400	IC	Organ-specific			No		Yes	(76) (6, 76, 80)

Other genes implicated in thymus development and as thymic transcription factors reviewed elsewhere: None morphogenic protein 4 (BMP4), Fibroblast growth factor 8 (Fgf8), Sonic hedgehog (shh), wingless-int 5n (Wnt5b, eyes absent homolog 1 (Eya1), homeobox protein a3 (Hoxa 3a), paired box protein (pax) 1 and pax9, sine oculis homolog 1/4 (Six 1/4), T-box 1 (Tbx1) (1).

Type of mutation: GOF, Gain of function; LOF: Loss of function.

Other cohorts with IFN-Abs: Psoriasis, pemphigus foliaceus, incontinentia pigmenti, myeloproliferative neoplasms, Graft-versus-host-disease after bone marrow transplantation, chronic viral hepatitis, severe COVID-19 including pneumonia, adverse reactions to yellow fever vaccine, healthy persons>70 years, effect of IFN-I treatment (34, 34, 81–92).

Headings: MI Mode of inheritance; IFN-I Systemic (or tissue fluid) Type I interferon signature; IFN-I Abs Type I interferon autoantibodies; AIRE Autoimmune Regulator.

Abbreviations genes/proteins: AIRE Autoimmune Regulator; FOXP3 forkhead box P3; NFKB2 nuclear factor kappa B subunit 2; RELB Proto-Oncogene, NF-KB Subunit; IKBKG inhibitor of nuclear factor kappa B kinase regulatory subunit gamma; NEMO NF-kappa-B essential modulator; IKK-γ inhibitor of nuclear factor kappa-B kinase subunit gamma; RAG recombination-activating gene; STAT signal transducer and activator of transcription; GOF gain of function; CTLA4 cytotoxic T-lymphocyte-associated protein 4 (CD152); IKZF IKAROS family zinc finger; LCK lymphocyte-specific protein tyrosine kinase; LAT Linker for activation of T cells; TNFAIP3 Tumor necrosis factor, alpha-induced protein 3; JAK3 Tyrosine-protein kinase janus.

Affected thymus: x: confirmed affected. (x) Most likely affected.

Abbreviations diseases: APS-I autoimmune polyendocrine syndrome type 1, IPEX Immunodysregulation, Polyendocrinopathy, Enteropathy, X-linked; DAVID; Deficient anterior pituitary with variable immune deficiency; BSS Bloch-Siemens syndrome; HP hypoparathyroidism; HA20 haploinsufficiency A20 disorder; A dysfunc. Adrenal dysfunction; SLE systemic lupus erythematosus; RA rheumatoid arthritis; fluct fluctuating disease; T1D Type 1 diabetes; MG myasthenia gravis; SCID severe combined immune deficiency; CVID common variable immunodeficiency; CID combined immunodeficiency; C.albicans Candida albicans.

Inheritance: AD; autosomal dominant; AR autosomal recessive, IC individual cases (most often); XLR X-linked recessive, XLD X-linked dominant.

Other marks: **¤**Only from case report (Sjøgren et al., 2022); **#**Name of disease and/or other manifestations than autoimmune and immune deficiency components; *In mice only; **^&^** IFN-I autoAbs in AIRE-deficiency and in MG/thymoma are mostly against the -ω and -α subtypes, but some reports have also found against IFN-β (22, 59, 93, 94). Empty: information not clear.

## Interferons are targeted by immune cells in individuals with diseases relating to the thymus

Thymic tolerance is crucial to avoid autoimmunity against TRAs and immune mediators, including the type I interferons (IFN-Is), which are special immune signals in viral host defense (100, 101) (see **Box 1**). There is a growing consensus that mutations in genes compromising thymic tolerance mechanisms might lead to a dysregulation of IFN-Is (22, 25, 27, 33, 38, 103–111). In the following text, examples of thymic abnormalities, dysregulation of IFN-Is, IFN-I autoAbs, and their link to immunomodulated diseases will be discussed.

Box 1 **Interferons**

In humans, there are three classes of interferons (IFNs); I (13 αs, one -ω, one -β, one -ϵ, one - к), II (IFN-γ) and III (four IFN-λs). Each class has its own preferred receptor; IFN-Is bind to the IFN-α receptor (IFNAR1/IFNAR2) while IFN-γ recognizes the interferon-gamma receptor (IFNGR1/IFNGR2) and the IFNLR1/IL10RB is the receptor for IFN-λs. The different IFN/receptor-complexes are activated via phosphorylation cascades involving diverse Janus kinases (JAKs)/Signal Transducer and Activator of Transcription 1 (STAT)/Tyrosine kinase 2 (TYK)-reactions, ultimately leading to translocation of the STATs into the nucleus and expression of interferon stimulated genes (ISGs) (see (102) for review). These IGSs can then mediate direct antiviral effects through viral RNA degradation and viral translation inhibition and by boosting adaptive responses. IFNs are therefore powerful molecules to protect tissues from invaders, but their activity must be tightly regulated to avoid excess activity and tissue damage.

The different IFN subclasses have a variety of distinct functions, but their biological activity overlaps to different extents causing a redundancy of the system. Type I IFNs (IFN-I) are especially important in antiviral host defense but also possess antitumor, and **anti**-proliferative effects. However, details regarding the specific functions within each subtype (e.g. the different IFN-αs compared to IFN-ω within IFN-Is) are still not clear.

An apt example of the relationship between thymus malfunction and IFN-I autoAbs is *AIRE* LOF mutations (112, 113). Patients with APS-I are found to have high titers of neutralizing IFN-I autoAbs (against the subtypes -αs and -ω) already at infancy, before clinical manifestations occur (109, 114, 115), and these autoAbs are remarkably stable throughout life. Even a partial loss of AIRE, as seen in dominant negative mutations, can induce development of autoAbs in affected individuals (116, 117). Intriguingly, serological autoAbs against IFN-Is are also found in several other diseases with decreased or complete loss of thymic AIRE expression, which may or may not implicate proper thymic architecture organization. Examples include patients with LOF mutations in the non-canonical Nuclear factor kappa-light-chain-enhancer of activated B cells (NF-KB) pathway (103, 105, 107, 118) (e.g. *NFKB2, RELB Proto-Oncogene, NF-KB Subunit (RelB), MAP3K14/NF-κB-inducing kinase (NIK)* and *IKBKG* (encoding NF-kappa-B essential modulator (NEMO) (34)) and hypomorphic *RAG1/2* mutations (37–41) (**Table 1**). In thymoma patients, a rare condition characterized by thymus carcinoma, thymopoiesis continues despite the lack of AIRE. This represents an example of acquired AIRE-deficiency and is also associated with anti-IFN-αs and -ω (59–61). The clinical consequences overlap in patients with thymoma and APS-I, with the appearance of autoimmune endocrine manifestations and failure to clear *Candida albicans* infections. Both conditions present with APS-I like antibodies, highlighting the importance of AIRE expression to ensure proper thymic function.

**Figure 1:**
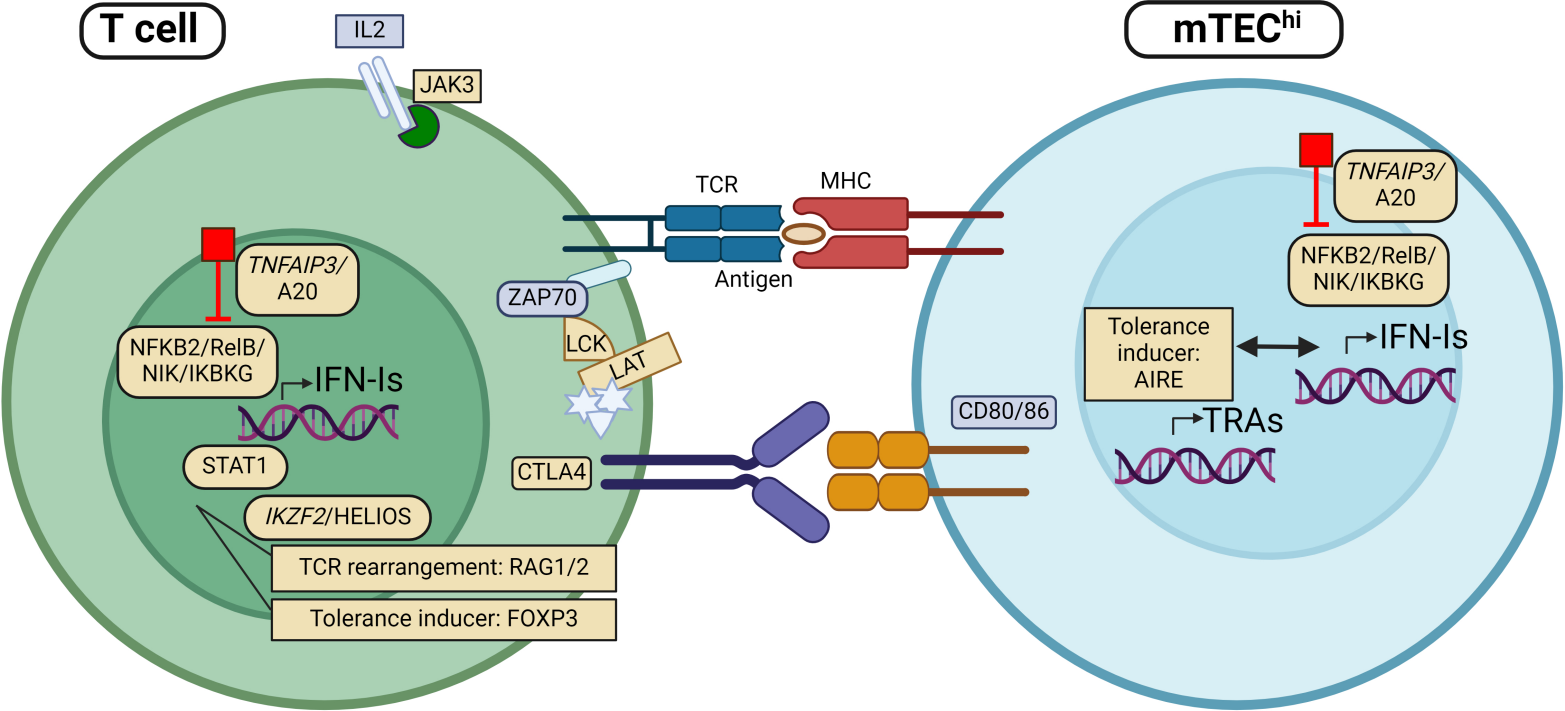
Patients with mutations in genes encoding proteins involved in regulation of type I interferon (IFN-I) expression, including *TNFAIP3, NFKB2, RelB, NIK* and *IKBKG*, have been found with autoantibodies against IFN-I (IFN-I autoAbs). The same applies to individuals with mutations in other genes implicated directly in thymic tolerance like *AIRE, RAG* and *FOXP3*. More recently, rare variants in genes encoding proteins with key functions in T cell regulation/activity, including *JAK3, STAT1, HELIOS, CTLA4, LCK* and *LAT* have been identified with IFN-I autoAbs. Genes (and proteins) that are found mutated in patients with IFN-I autoAbs are marked in yellow. APC, Antigen presenting cell; mTEC, medullary thymic epithelial cells; TCR, T cell receptor; MHC, major histocompatibility complex. Figure created with BioRender.com.

Interestingly, sporadic cases with other mutations located in immune regulatory genes have also been found to acquire anti-IFN-I autoAbs. This includes *STAT1* gain of function (GOF) mutations and rare (presumably LOF) variants in *CTLA-4, IKZF2, FOXP3, LCK, LAT, TNFAIP3* and *JAK3* (25, 27, 38, 105)(**Table 1**). Their encoded proteins are important in T cell activity and for the generation of central T cell tolerance in the thymus (**Table 1**, **Figure 1**). Although the physiological roles of these autoAbs is yet unknown, they serve as excellent markers of disease, suggesting that genomic analysis should be performed when detected in patients (27).

## IFN-I autoAbs in other disease cohorts provide clues on their etiology and immune consequences

There is strong evidence that overproduction of IFN-Is is associated with pathological roles in several systemic (119, 120) and organ-specific autoimmune disorders (121–126). Even when administered as treatment of chronic active hepatitis, IFN-α may cause hyperthyroidism or autoimmune hepatitis (127–129). Several primary immune deficiencies, caused by mutations in genes directly involved in regulation of IFN-I responses, also present with dysregulated IFN-I profiles (130). However, these disorders rarely come with IFN-I autoAbs as long as thymic immune tolerance is intact (111) (**Table 1**). Very high levels of IFN-Is and/or chronic inflammation might still break peripheral immunological tolerance, and the first stories on IFN-I autoAbs were reported in various patient cohorts treated with IFN-β (131–133). A sudden endogenous increase of cytokine expression following e.g. septic shock or viral (e.g. severe COVID-19, acute viral hepatitis and chronic HIV) or bacterial infections have also been reported to induce anti-cytokine autoimmunity (reviewed elsewhere (134)). IFN-I autoAbs were subsequently identified in patients with chronic graft-versus-host disease following allogeneic bone marrow transplantation (81, 82), in pemphigus foliaceus (83) and further in cohorts of SLE patients (67, 135). The IFN-I autoAbs are less prevalent in other autoinflammatory disorders like Sjøgren’s disease (SS) and rheumatoid arthritis (RA), where reported frequencies depend on the cohorts, subtypes of disease, IFN-I autoAbs assays and “threshold level” (67, 135). The likelihood of developing IFN-I autoimmunity follows a spectrum from ~100% in AIRE deficiency, to various, but lower, frequencies in other genetic disorders that affect the development and function of the thymus, and as also reflected by dominant AIRE mutations Patients with systemic autoimmune disorders probably need additional environmental triggers, such as infections, drugs or vaccines, to develop IFN-I autoimmunity.

## Recognized physiologic roles of autoAbs to IFN-Is

Recent studies have highlighted the role of IFN-I autoAbs in relation to the outcome of infectious diseases, such as COVID-19. Early in the COVID-19 pandemic, it was reported that patients with APS-I became critically ill upon being infected with the SARS-CoV2 virus (136–138). Pre-existing IFN-I autoAbs was the common denominator and it was established that the viral defense was affected in these patients. Although the severity of COVID-19 disease in APS-I has later been challenged (139, 140), perhaps as a consequence of evolving virus variants and/or vaccination, the fact that individuals with anti-IFN-I autoAbs are at risk of severe lung manifestations still stands. This is supported by the presence of high levels of neutralizing IFN-I autoAbs in about 10% of patients across different age groups suffering from life-threatening COVID-19 (34, 137). Later it was shown that neutralizing IFN-I autoAbs were present in serum from about 4% of uninfected individuals over 70 years of age, suggesting that thymic involution might play a role in initiating IFN-I autoimmunity (84). The host protection against other pathogens like herpes zoster and/or varicella pneumonia has also recently been found to be impaired in different patient cohorts with serological anti-IFN-I autoAbs like APS-I, Omenn’ syndrome and SLE (68, 141–143). Adverse reactions causing life-threatening disease following vaccination with a yellow fever live attenuated vaccine were furthermore recently noted with post-findings of IFN-I autoAbs in otherwise healthy individuals with a large age span (13-80 years) (85). These autoAbs were shown to inhibit the protective effect of IFN-Is against strains of the yellow fever vaccine in subsequent experiments (85). This indicates that IFN-I autoAbs can underlie development of critical disease following live virus vaccines in persons with immune deficiencies and/or known presence of IFN-I autoAbs. A systemic effect of presence of IFN-I autoAbs resulting in a compromised host immune efficacy is also indicated by the correlation of IFN-I autoAbs in APS-I patients (and others with high levels of neutralizing IFN-I autoAbs) with reduced peripheral expression of interferon stimulated genes (ISG) (20, 21, 92). However, it has also been suggested that these autoAbs can protect against type 1 diabetes (T1D) and thyroid disease, as reported in a few APS-I patients without IFN-α autoAbs (144, 145). The possible protective role of IFN-I autoAbs in distinct biological processes in inflammatory disorders is supported by the finding of an association of IFN-α autoAbs with decreased/normalized ISG profiles and fluctuating disease activity in the systemic autoimmune disorders SLE, SS and RA (67, 135, 146). These observations have led to the application of molecules of the IFN-I pathway as new therapeutic targets in subgroups of SLE (86–88), e.g. anifrolumab which blocks the activity of the IFN-I receptor. Notably, as IFN subtypes are redundant in their properties, untargeted IFN-I subtypes (which are not attacked by the hosts immune system in susceptible patients) may compensate and limit the biological consequences of IFN-I autoimmunity.

## Thymus is involved in regulation of IFN-I tolerance

Thymic mechanisms are challenging to study in humans due to the inaccessibility and shrinking of the organ with age (involution). Therefore, most knowledge comes from mouse studies. Over the past years, it has become clear that Ifn-Is in mice are expressed at high levels in the thymus, and that Aire in mTECsis involved, either directly or indirectly, in regulating their expression (100, 147–149). Subsets of developed “post-/late-Aire+” mTECs produce relatively high amounts of Ifn-β at homeostatic conditions, and the Ifnα receptor is expressed by both thymocytes and antigen-presenting cells in the thymus, implicating that these cells have the potential to respond to local Ifn-I signaling. It is still unclear whether the Ifn-β expression is directly linked to the transcriptional activity of Aire, but studies have shown that knocking out Aire in mice leads to the loss of Ifn-β signals in the thymus (100, 150). This indicates that Ifn-Is can be direct responders of Aire’s transcription factor activity or that Aire can affect Ifn-I biology by its function in thymic architecture and thymopoiesis. Furthermore, as tonic Ifn-α expression has not been detected in mice thymi (100), there must be considerable differences in expression and mechanisms of tolerance achievement between the Ifn-I subtypes (151–153). To complicate the picture, Nf-kb recruitment is necessary for both Aire induction and as a sensor of viral exposure leading to activation of an Ifn-I immune response (100, 154, 155). Hence, Aire and Ifn-Is, at least Ifn-β, are connected by the Nf-kb pathway. IFN-I regulation is even more complex in humans than in mice, with additional IFN-α subtypes and IFN-ω (which is lacking in mice). In inborn or acquired AIRE deficiency, seen in APS-I patients and myasthenia gravis (MG) patients with thymoma (156, 157) respectively, expression of IFN-I will be out of control, leading to dysregulation of the feedback loop and probably high local levels of these cytokines in the thymus (156). The lack of AIRE will simultaneously interfere with proper immune tolerance against AIRE-regulated TRAs, likely also affecting IFN-I expression (100), leading to autoreactivity against them. The devastating combination of high local levels of IFN-Is with no generated immune tolerance might then become a self-driving loop continuing when the autoimmune naïve T cells exit the thymus. For MG with thymoma, further evidence for a link between AIRE and IFN-Is has been established as the expression of the α-subunit of the acetylcholine receptor (AChR), the main autoantigen in MG, is regulated by both AIRE and IFN-β in the thymus (155, 158). A plausible explanation for MG in some thymoma patients may then be a “bystander effect” where neoplastic cells fail to drive AChR and IFN-I generation in an AIRE free milieu, breaking tolerance against them, causing both anti-IFN and anti-AChR autoimmunity. Upregulation of IFN-Is is also responsible for the generation of anti-AChR autoAbs in early onset MG without thymoma. This is shown indirectly as upregulation of IFNs increases B cell infiltration, ectopic GC formation and ISG stimulation. However, patients with early onset MG without thymoma do not have anti-IFN-Is, despite having a high-IFN-I ISG profile (60), probably because of an intact tolerance mechanism. This starts a vicious circle where upregulation of IFN-Is again exaggerates ectopic B cell formation in the thymus of these patients. Regulatory T cells (Tregs) have also been studied in MG-patients in relation to IFNs. Of interest, IFN-I expression in Tregs may affect the development of these natural suppressor cells (159, 160) and IFN-I signaling within Tregs promotes their inherent suppressive role in anti-viral defense (161). It further protects the Treg population under inflammation (162). Several of the other conditions with IFN-autoAbs also have Treg disturbances (**Table 1**), but the role of the IFN-Treg symbiosis must be studied further before causative/functional links are fully established.

Indirect proof for the importance of the thymus in IFN tolerance is also the very concept of this article: the abundance of autoAbs against IFN-Is in AIRE deficiency, in defects of the NF-KB axis (27, 34) and in defects of genes encoding key factors of immune tolerance, like FOXP3, RAG and CTLA-4 (**Table 1**). There is, however, yet one big question left to answer: Why are the other type I IFNs (IFN-β, -ϵ, and –к) seldom targets of autoimmunity? One theory is that mechanisms that promote IFN-I production in mTECs are also likely to be induced in tolerance to IFNs and ISG, and type, timing, and expression levels of IFN-I is vital in thymopoiesis. When first targeted, IFN-Is can be implicated in thymic atrophy leading to the loss of developing thymocytes with increased survival of self-reactive cells (modulated by IFN-β) (163–165). IFN-Is also affect thymocyte selection and maturation, and development/maturation of mTECs are required for tolerance against these immune modulators (147, 151).

We hypothesize two, not mutually contradictory, alternative mechanisms for the generation of IFN-I autoAbs. (I) T cell driven hypothesis. In the thymus, IFNs are expressed by mTECs and AIRE is directly involved in their production and/or in the development of mTECs (100, 166). When thymic organogenesis or AIRE expression fails, tolerance against IFNs is disturbed, leading to autoimmunity towards IFN-I; (II) B cell driven hypothesis. High thymic and/or tissue levels of IFNs in affected individuals feed into the infectious environment of the organs in early development and could kickstart tissue cell destruction with autoimmune mechanisms. The initial cause of IFN-I secretion may be a virus or an unknown environmental trigger. When the IFNs reach high levels in a “still not achieved immune tolerance” situation, this will mediate an autoimmunization environment in both the thymus and other organs/periphery, resulting in peripheral break of tolerance. Then, instead of a direct break of tolerance, the immune system reacts by feedback loops when identifying the dangerous concentrations of cytokines in the wrong place at the wrong time, thereby stimulating the adaptive immune cells to start producing antibodies against IFNs to fight the uncontrolled situation. Either way, when tolerance against anti-IFNs is broken, this establishes an anti-IFN response that is maintained throughout life.

## Conclusion

Defining the molecular mechanisms behind thymus-associated and/or IFN-I linked disorders has revolutionized our understanding on IFN-I regulation and messaging. New evidence of anti-IFN-I interference with viral disease outcome should warrant analysis of anti-IFN-Is in individuals with known autoimmune and/or immunodeficiency disorders before giving advice on vaccine modalities for disease prevention and before administrating treatment with IFN-α or -β for certain disorders. Furthermore, as it has become clear that anti-IFN-Is are often connected with inborn or acquired immunodeficiency and thymic disease, sequencing of positive IFN-I autoAbs cases should be considered to identify their underlying molecular cause.

## Author contributions

BO: Conceptualization, Writing – original draft, Writing – review & editing. TS: Conceptualization, Writing – original draft, Writing – review & editing. AW: Conceptualization, Writing – original draft, Writing – review & editing.
